# Contracted or Vanishing Gallbladder: A Case Report

**DOI:** 10.7759/cureus.84382

**Published:** 2025-05-19

**Authors:** Karthika PS, Sundeep Selvamuthukumaran, Pola Govardhan Kumar, BV Sreedevi, Ankita Swarnkar

**Affiliations:** 1 General Surgery, Sree Balaji Medical College and Hospital, Chennai, IND; 2 General Surgery, Sri Ramaswamy Memorial (SRM) Medical College Hospital and Research Centre, Chennai, IND

**Keywords:** case report, cholangitis, cholecystitis, choledocholithiasis, contracted gallbladder, liver cirrhosis, obstructive jaundice, vanishing gallbladder

## Abstract

A contracted or "vanishing" gallbladder is a condition characterized by severe atrophy or fibrosis of the gallbladder, often resulting from chronic inflammation, recurrent cholecystitis, gallstone disease, or metabolic disorders such as diabetes and chronic alcohol use. The etiopathogenesis involves progressive fibrosis due to persistent biliary obstruction, chronic infection, or impaired blood flow, particularly in patients with liver cirrhosis. While the exact incidence remains unclear, it is more prevalent in individuals with long-standing hepatobiliary diseases or metabolic syndromes. Common symptoms include severe right upper quadrant pain, jaundice, nausea, vomiting, and signs of obstructive jaundice, such as dark urine and pale stools. Diagnostic investigations typically involve ultrasound, contrast-enhanced computed tomography (CECT), magnetic resonance cholangiopancreatography (MRCP), and endoscopic retrograde cholangiopancreatography (ERCP) to assess biliary obstruction, liver pathology, and gallbladder morphology. Treatment requires a multidisciplinary approach, including initial ERCP for biliary decompression, followed by surgical intervention (laparoscopic or open cholecystectomy), with conversion to open surgery often necessary due to dense adhesions and fibrosis. Postoperative care focuses on managing comorbidities, preventing complications, and long-term monitoring of liver health.

This case report presents the challenging management of a 34-year-old male with a history of diabetes mellitus, chronic alcohol use, and liver cirrhosis, who presented with severe right upper quadrant pain, jaundice, and ascites. Initial evaluation revealed cholangitis, calculous cholecystitis with choledocholithiasis, and imaging findings consistent with liver cirrhosis. The patient underwent ERCP for biliary stenting and sludge extraction, followed by an attempted laparoscopic cholecystectomy. Intraoperatively, dense adhesions and fibrosis obscured the gallbladder, confirming a contracted or "vanishing" gallbladder, prompting conversion to an open procedure. The case highlights the diagnostic complexities of contracted gallbladders, which often result from chronic inflammation, fibrosis, or metabolic disorders. Preoperative imaging, including ultrasound and MRCP, played a critical role in identifying biliary obstruction and liver pathology. However, intraoperative findings necessitated adaptive surgical decision-making to mitigate risks such as bile duct injury or hemorrhage. The patient’s multiple comorbidities further complicated management, emphasizing the need for a multidisciplinary approach involving gastroenterologists, surgeons, and hepatologists. Postoperative recovery was closely monitored for complications, including infection and bile leaks. The patient was discharged with follow-up care focusing on liver health, diabetes management, and alcohol cessation. This report underscores the importance of thorough preoperative assessment, flexibility in surgical technique, and collaborative care in optimizing outcomes for patients with complex gallbladder pathology. Future research should explore long-term outcomes and improved imaging techniques to enhance surgical planning for such challenging cases.

## Introduction

The gallbladder serves a vital function in bile storage and concentration, enabling efficient fat digestion and absorption [1]. Pathological conditions such as acute cholecystitis, choledocholithiasis, and cholangitis frequently require surgical management, with laparoscopic cholecystectomy remaining the preferred approach [2]. However, the contracted or "vanishing gallbladder" - characterized by severe fibrosis and anatomical distortion - presents significant diagnostic and operative challenges [3]. These complex cases typically result from chronic inflammation, metabolic disorders including diabetes mellitus, or advanced liver cirrhosis, all of which contribute to structural and functional gallbladder deterioration [4]. While preoperative imaging modalities like ultrasonography and magnetic resonance cholangiopancreatography (MRCP) are essential for surgical planning, intraoperative findings often necessitate procedural modifications, including conversion to open cholecystectomy [5]. This case report examines a 34-year-old male with multiple comorbidities, emphasizing the critical role of comprehensive clinical evaluation, advanced imaging interpretation, and multidisciplinary collaboration in managing contracted gallbladder pathology. The discussion highlights the importance of surgical adaptability and meticulous postoperative care to minimize complications such as bile duct injury or hemorrhage while simultaneously addressing underlying systemic conditions including cirrhosis and metabolic dysfunction.

## Case presentation

A 34-year-old male, presented to the emergency department with a one-week history of severe right upper quadrant pain described as sharp and radiating to the back. He reported worsening abdominal distension noted by both himself and family members, along with visible jaundice characterized by yellowing of the sclera that prompted his hospital visit. Additional symptoms included high-colored urine indicative of bilirubin excretion and pale stools suggestive of obstructive jaundice. The patient reported two episodes of fever two weeks prior to presentation. Notably, there was no associated nausea, vomiting, melena, hematemesis, hematochezia, constipation, or diarrhea.

The patient's past medical history was significant for multiple comorbidities including type 2 diabetes mellitus diagnosed 10 years prior with suboptimal glycemic control, systemic hypertension managed with antihypertensive medications, and a history of acute calculus cholelithiasis treated conservatively in 2018. His cardiovascular history included coronary artery disease status post angioplasty in 2018 with ongoing antiplatelet therapy. Other notable conditions included dyslipidemia, chronic hepatitis B infection (HBsAg positive), and a 20-year history of chronic alcohol use. No significant family history of liver or gallbladder disease was reported.

On physical examination, the patient appeared icteric with stable vital signs (blood pressure (BP) of 130/85 mmHg, heart rate (HR) of 85 beats per minute (bpm)) but exhibited tachypnea (30 breaths/min). Abdominal examination revealed mild distension with a positive Murphy's sign. Palpation demonstrated tenderness in the right hypochondrium with guarding but no rigidity. Percussion detected minimal ascites with shifting dullness, and bowel sounds were present though diminished in intensity.

Initial laboratory investigations showed mild leukocytosis suggesting infection, while liver function tests revealed elevated total bilirubin with direct bilirubin predominance, alkaline phosphatase, and mildly elevated aspartate aminotransferase (AST) and alanine aminotransferase (ALT). Pancreatic enzymes were notably elevated, raising concern for pancreatitis or biliary obstruction (Table 1).

**Table 1:** Laboratory test results

Test	Patient's value	Normal reference range
White blood cells (WBC)	12,000/µL	4,000-11,000/µL
Total bilirubin	5.2 mg/dL	0.1-1.2 mg/dL
Direct bilirubin	3.1 mg/dL	0.0-0.3 mg/dL
Alkaline phosphatase (ALP)	240 U/L	30-120 U/L
Aspartate aminotransferase (AST)	80 U/L	10-40 U/L
Alanine aminotransferase (ALT)	60 U/L	7-56 U/L
Amylase	334 U/L	30-110 U/L
Lipase	380 U/L	10-140 U/L

**Figure 1:**
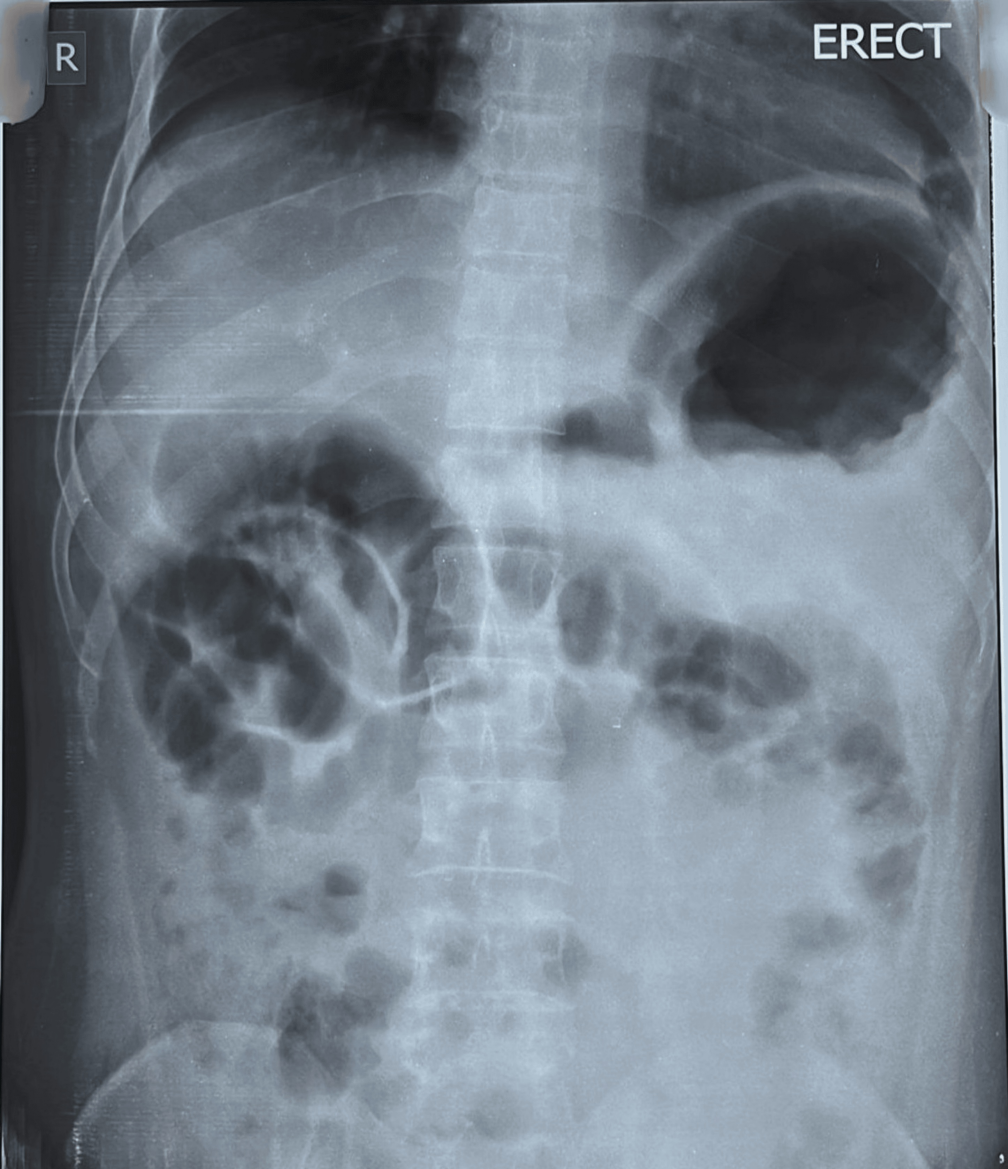
Erect abdominal X-ray showing no signs of obstruction

Imaging studies began with an erect abdominal X-ray which showed no signs of obstruction (Figure 1).

**Figure 2:**
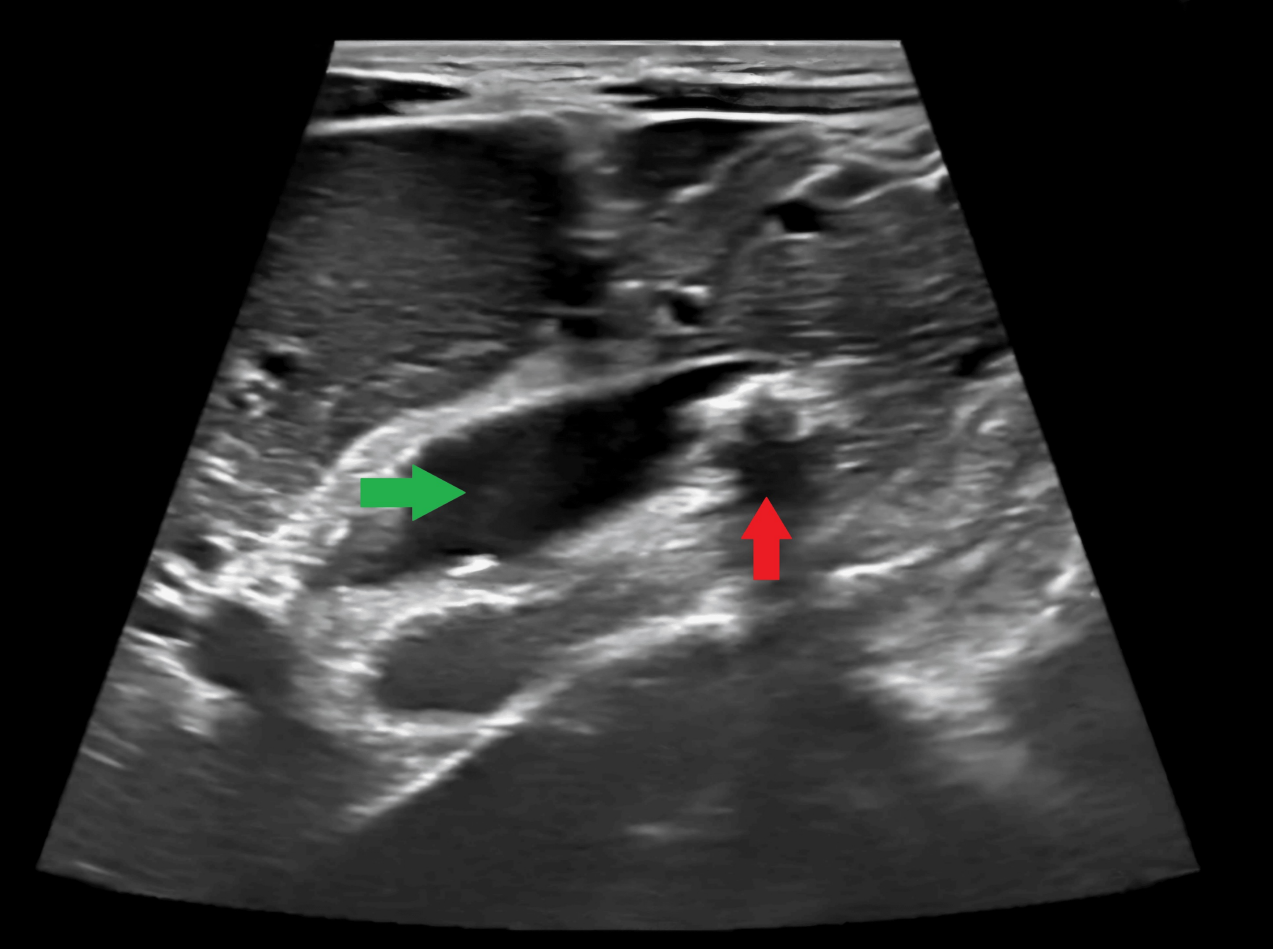
Ultrasound of abdomen showing calculus at the distal common bile duct causing proximal bile duct dilatation. The green arrow shows the common bile duct, and the red arrow shows the calculi

Abdominal ultrasound demonstrated early liver parenchymal disease, a 2 × 2 cm calculus at the distal common bile duct (CBD) causing proximal bile duct dilatation, a contracted gallbladder, and mild ascites (Figure 2).

**Figure 3:**
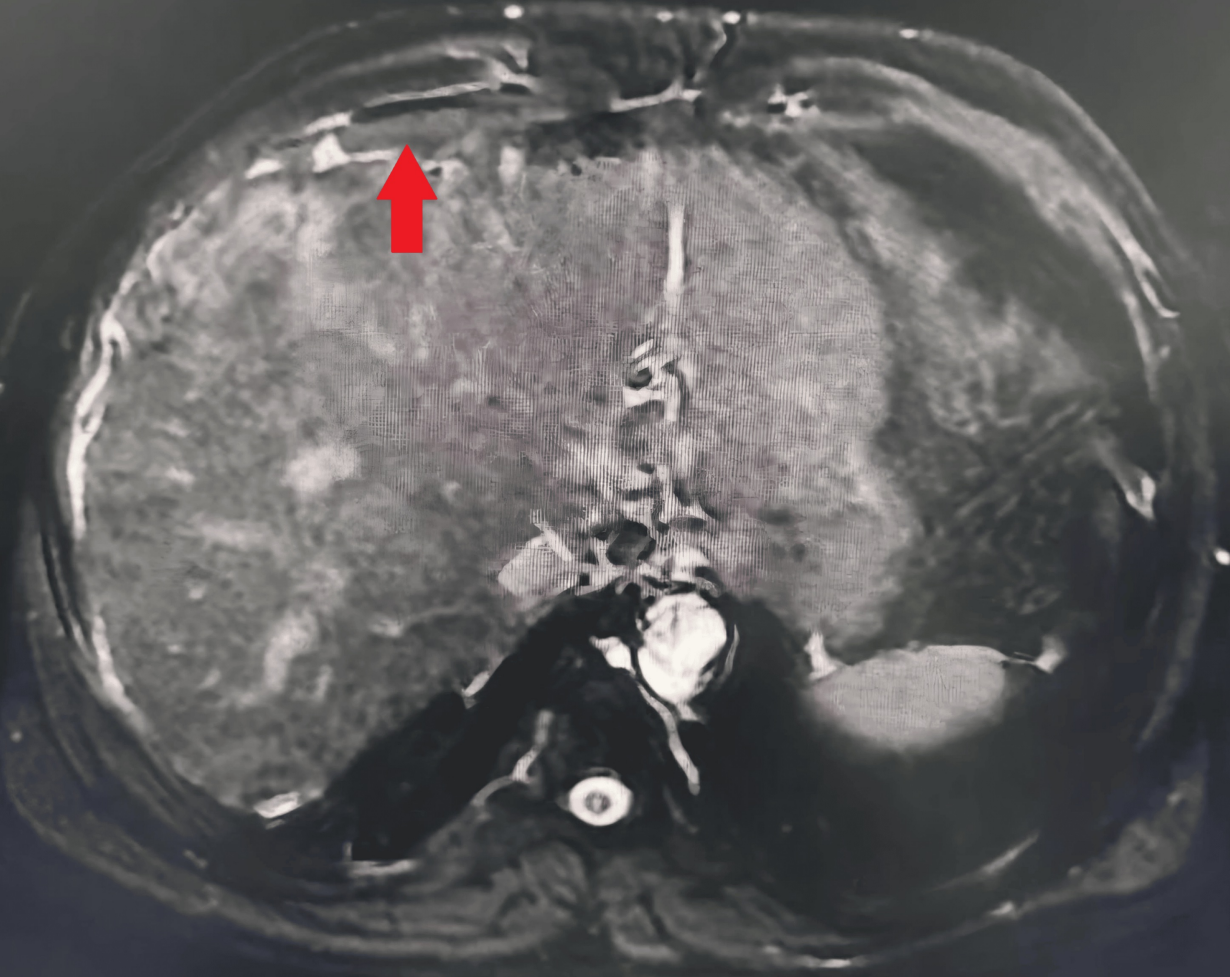
Magnetic resonance cholangiopancreatography (MRCP). The red arrow shows nodularity and irregular contours of liver surface

**Figure 4:**
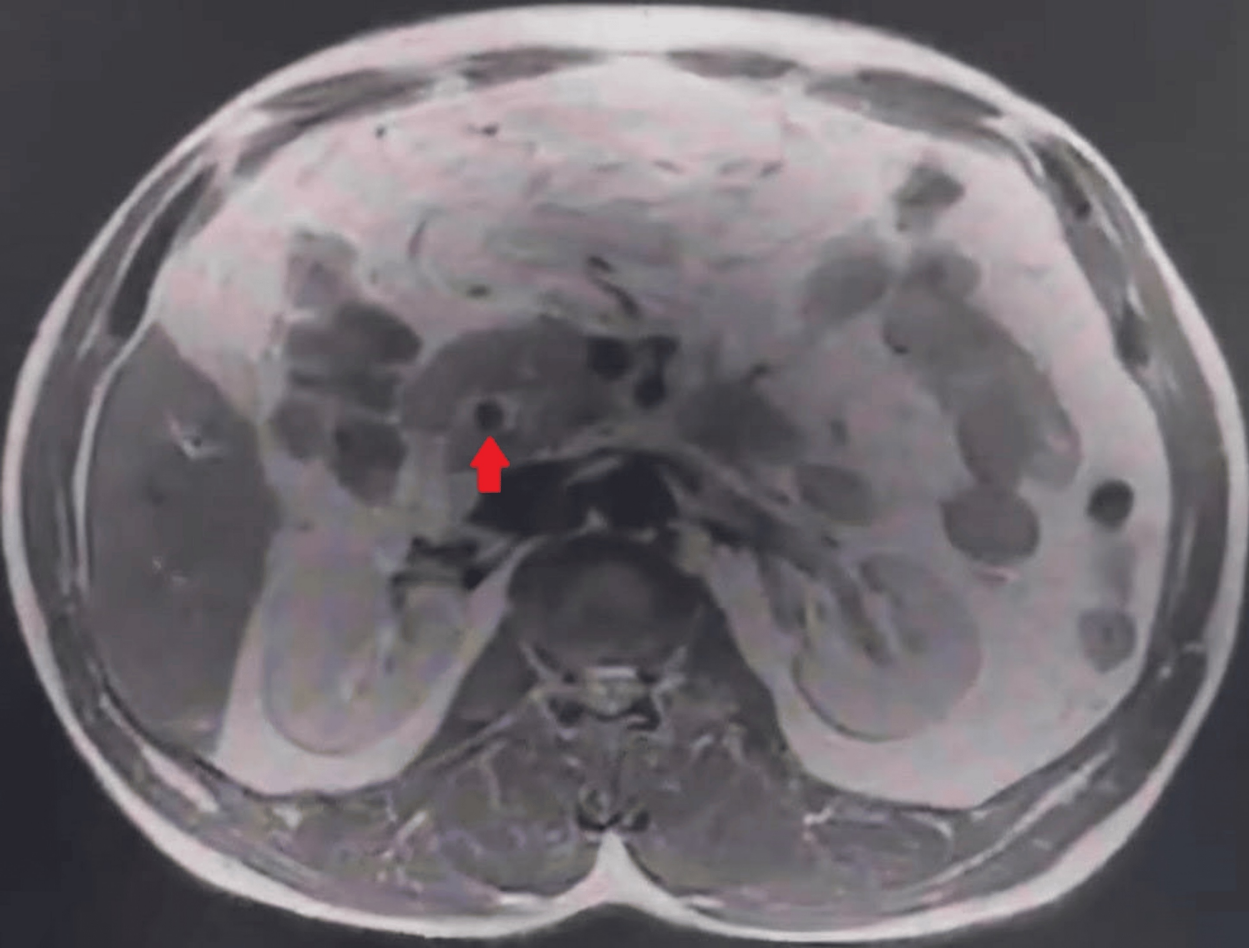
Magnetic resonance cholangiopancreatography (MRCP). The red arrow shows choledocholithiasis (gallstones within the common bile duct)

MRCP further characterized the liver cirrhosis with nodularity and irregular contours, along with a dilated CBD containing multiple stones and a contracted gallbladder complicating potential laparoscopic access (Figures 3-4).

**Figure 5:**
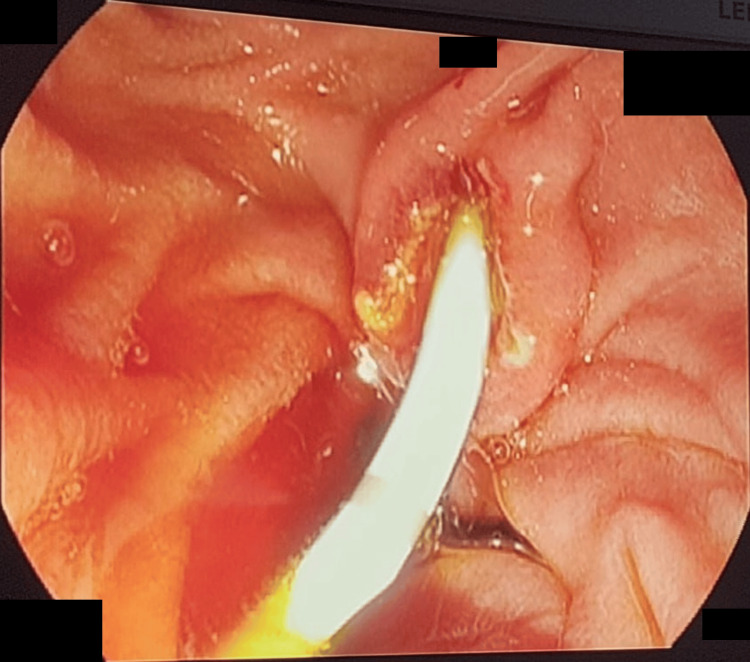
Endoscopic retrograde cholangiopancreatography (ERCP) showing common bile duct dilation with sludge but no large obstructing stones, leading to successful biliary sphincterotomy with sludge extraction

**Figure 6:**
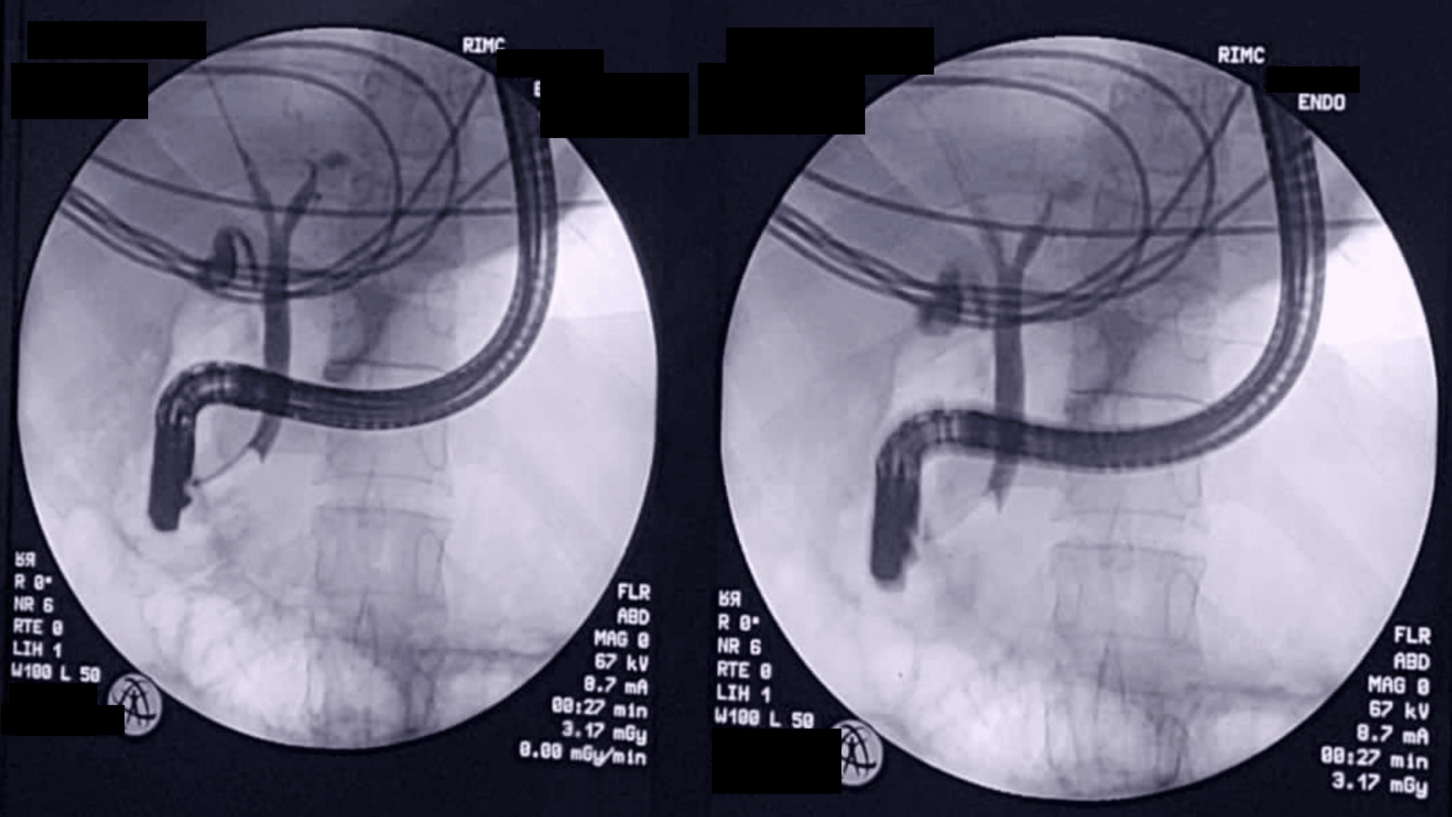
Endoscopic retrograde cholangiopancreatography (ERCP) showing biliary stenting (common bile duct plastic stenting)

Subsequent endoscopic retrograde cholangiopancreatography (ERCP) confirmed CBD dilation with sludge but no large obstructing stones, leading to successful biliary sphincterotomy with sludge extraction and biliary stenting (Figures 5-6).

**Figure 7:**
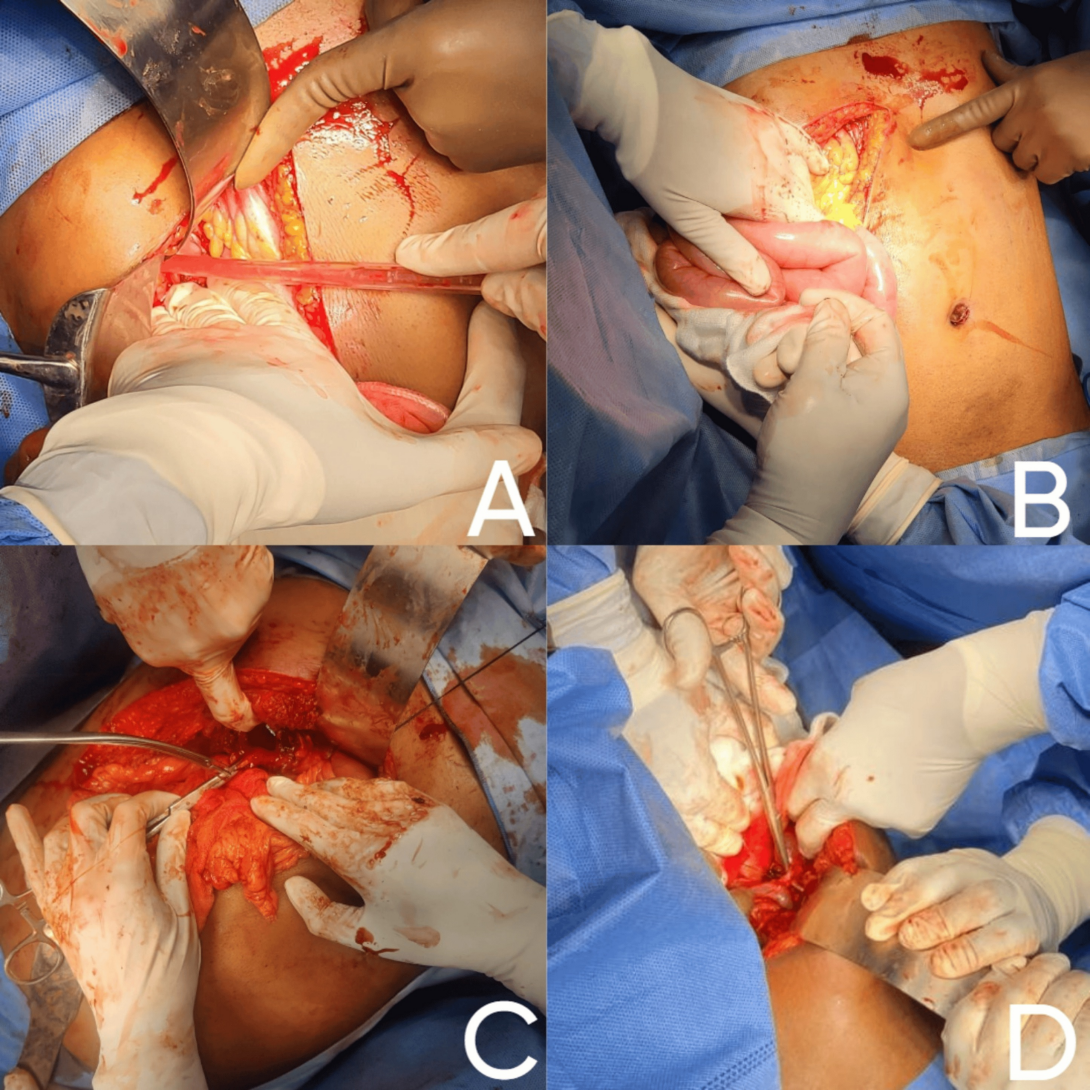
Intraoperative images A) The right subcostal Kocher's incision was made. The abdomen opened in layers. The liver is retracted using a Deaver retractor. B) Dilated bowel loops; adhesions covering gallbladder fossa. C) Adhesiolysis, only the gallbladder fundus could be visualized. D) Contracted intrahepatic gallbladder fundus opened.

The patient's initial management included intravenous fluids, broad-spectrum antibiotics for cholangitis, and opioid analgesics for pain control. Surgical intervention with attempted laparoscopic cholecystectomy was complicated by severe fibrosis and dense adhesions obscuring the gallbladder and dilated bowel loops, necessitating conversion to an open procedure. Intraoperative findings included significant scarring from previous inflammation and multiple black stones in the gallbladder bed. Only the gallbladder fundus was visualized, and the intrahepatic gallbladder was contracted. Gallbladder stones were retrieved. The anterior wall of the gallbladder was excised and sutured. Subtotal cholecystectomy was done (Figure 7).

**Figure 8:**
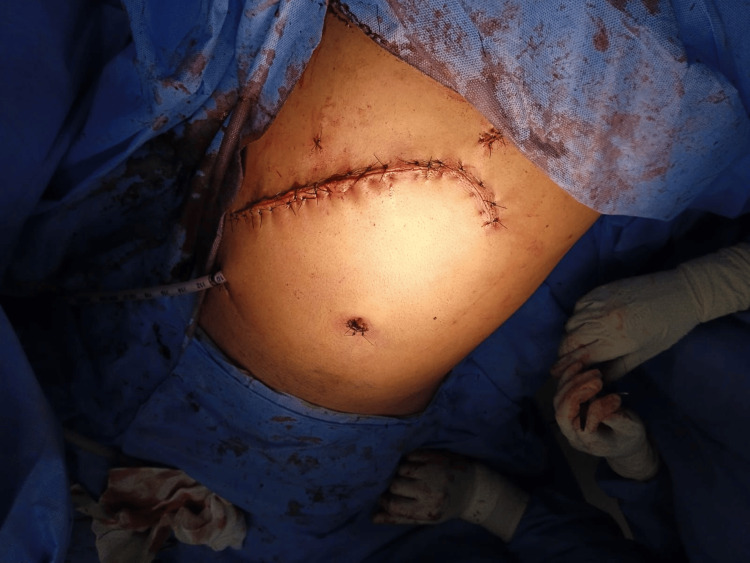
Postoperative on-table image with intercostal drain (ICD) drain

An intercostal drain (ICD) was placed and the abdomen was subsequently closed in layers (Figure 8).

**Figure 9:**
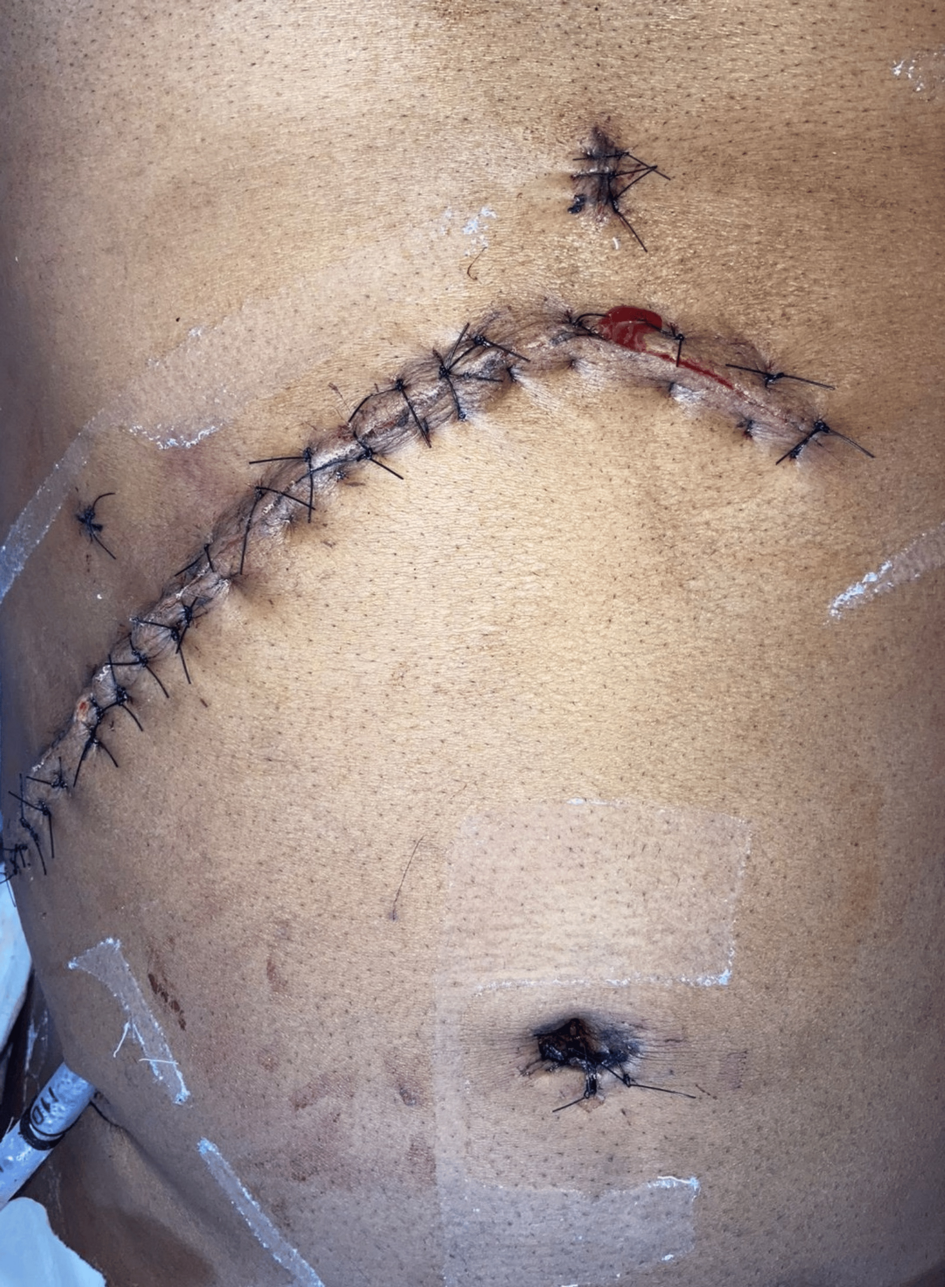
Tenth postoperative day follow-up imaging prior to suture removal

**Figure 10:**
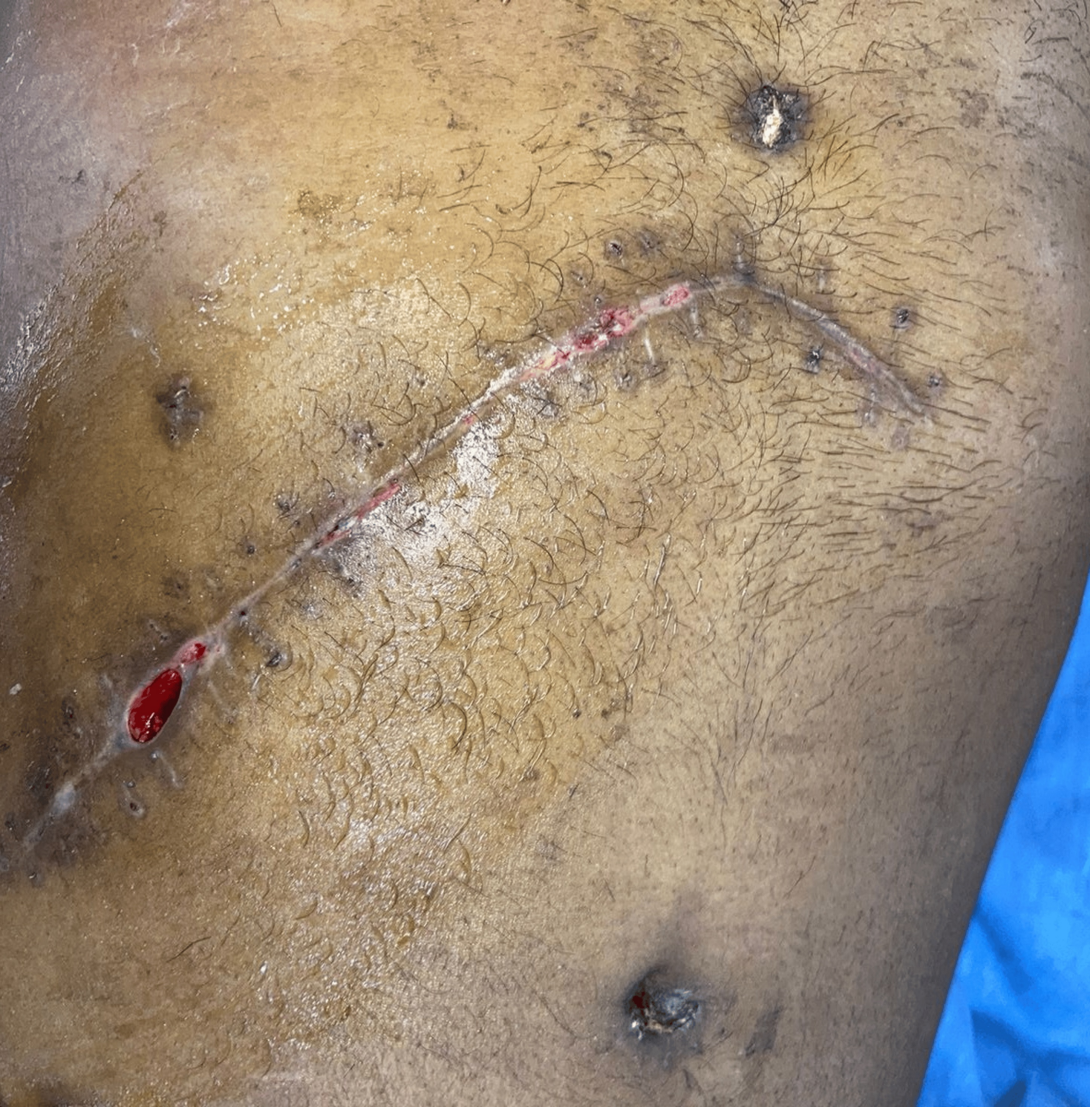
Thirtieth postoperative day follow-up imaging after suture removal

The postoperative period was uneventful. The patient was started on appropriate intravenous fluids, antibiotics, analgesics, and other supportive measures. Ryle's tube (RT) was inserted. The patient gradually resumed oral intake, with successful recovery, and was discharged. The patient was on regular follow-up (Figures 9-10).

## Discussion

The contracted gallbladder is a condition characterized by reduced size and function, which may result from chronic inflammation, repeated episodes of cholecystitis, or gallstone disease [6], with patients exhibiting chronic liver disease and portal hypertension being particularly susceptible to gallbladder atrophy due to impaired blood flow and biliary drainage [7]. The etiology is complex and multifactorial, involving chronic cholecystitis leading to fibrosis and scarring [8], nutritional factors like chronic alcohol use (as seen in this case) causing liver dysfunction and reduced motility [9], and metabolic conditions such as diabetes impacting gallbladder function and morphology [10]. While laparoscopic cholecystectomy is generally preferred for its advantages of lower morbidity, faster recovery, and reduced postoperative pain [11], cases with contracted gallbladders or significant inflammation often require conversion to open procedures due to challenges in visualization and access [12], with intraoperative decision-making being crucial to mitigate risks of bile duct injury, hemorrhage, or incomplete gallbladder removal, where surgical experience and adaptability significantly influence outcomes [13].

This case underscores the necessity of a multidisciplinary approach involving gastroenterologists, surgeons, and internists for optimal preoperative assessment, liver function monitoring, and timely management of complications like cholangitis or pancreatitis [14]. Postoperative recovery requires vigilant monitoring for potential complications, including surgical site infections (necessitating antibiotics), bile leaks (requiring drainage or reintervention), and intra-abdominal hemorrhage (potentially needing reoperation), particularly in patients with comorbidities like liver disease, with this patient being discharged after one week with instructions for liver function monitoring, infection surveillance, and lifestyle modifications, including dietary changes and alcohol cessation. Differential diagnosis must carefully consider acute cholecystitis (gallbladder inflammation from stone obstruction, supported by history and imaging), choledocholithiasis (CBD stones causing obstructive jaundice, evident on imaging), cholangitis (bile duct infection confirmed by clinical and laboratory findings), obstructive jaundice (secondary to biliary obstruction), and liver cirrhosis (with nodularity on imaging), as accurate differentiation is essential for appropriate treatment given the frequent symptom overlap that can complicate clinical assessment. This comprehensive understanding of contracted gallbladder pathology, from etiology through postoperative management, highlights the importance of thorough evaluation, adaptive treatment strategies, and multidisciplinary collaboration in optimizing patient outcomes.

## Conclusions

This case highlights the complexities associated with diagnosing and managing a contracted gallbladder in the context of multiple comorbidities. A tailored approach, including thorough investigations and consideration of the patient's overall health status, is essential for optimal outcomes. The necessity for a multidisciplinary team is paramount in navigating the challenges presented by such complex cases.
